# Bridging the Divide between Science and Journalism

**DOI:** 10.1186/1479-5876-8-25

**Published:** 2010-03-10

**Authors:** Laura Van Eperen, Francesco M Marincola, Jennifer Strohm

**Affiliations:** 1 Van Eperen & Company, Strategic Communications Consulting, Bethesda, MD 20817, USA; 2 Infectious Disease and Immunogenetics Section (IDIS), Department of Transfusion Medicine, Clinical Center, National Institutes of Health, Bethesda, Maryland, 20892, USA

## Abstract

There are countless reasons nearly every scientist should learn how to communicate effectively with the media, including increased understanding of critical research findings to attract or sustain funding and build new professional partnerships that will further propel forward research. But where do scientists begin? *Bridging the Divide between Science and Journalism *offers practical tips for any scientist looking to work with the media.

Given the traditional and internet-based sources for medical research and healthcare-related news now available, it is imperative that scientists know how to communicate their latest findings through the appropriate channels. The credible media channels are managed by working journalists, so learning how to package vast, technical research in a form that is appetizing and "bite-sized" in order to get their attention, is an art. Reducing years of research into a headline can be extremely difficult and certainly doesn't come naturally to every scientist, so this article provides suggestions on how to work with the media to communicate your findings.

## Bridging the Divide between Science and Journalism

With http://WebMD.com, http://healthline.com, http://DiagKNOWsis.org and numerous medical consumer websites now available, more individuals are relying on them, and the evening network news for the latest media headlines to educate and guide them in their medical decisions. Now more than ever, it is important for scientists and journalists to bridge the communication divide that exists between them [1]. In doing so, scientists will not only be able to assist the public in making better informed decisions about their healthcare, but also personally reap the benefits of increased funding for their research, enhanced career opportunities and improving the chances for further scientific breakthroughs across disciplines.

Many reading this article may have already had an experience working with a journalist covering their research. In the professional communications realm, it is frequent that individuals have had favorable and not so favorable experiences with the media. With scientists, it tends to be the latter for several reasons.

First, because research often has many detailed nuances and the media don't have the time or the space to cover all of those points. The length of the average evening news story is 70 seconds. Print stories can range anywhere from 100 word briefs to 1000 word articles, with the latter becoming more and more scarce. Therefore, the format of much of today's news coverage simply doesn't allow for detailed reporting.

Second, it can be difficult for scientists and journalists to communicate with each other because often they speak in terms the other doesn't understand. More than ever, journalists must know a little about a lot of things. They typically cover a wide variety of topics on very short deadlines. If a topic is too complex, it will simply be lost in the shuffle of the other hundreds of e-mails, phone calls and information they are inundated with on a daily basis.

These issues, coupled with the general public's (the media's readers/viewers/listeners) very limited understanding of basic science, can make it extremely difficult for scientists to get their points across in the media. In fact, a 1997 National Science Foundation study found that half the American public doesn't know that it takes a year for the Earth to rotate around the sun [2]. If Americans have difficulty recalling that simple fact, why would we expect them to understand the complexities of scientific research and its latest discoveries?

Most journalists fall into this group too. The overwhelming majority of scientists surveyed in a First Amendment Center, Freedom Forum study felt that few in the media understand the nature of science and technology, with 72 percent saying that journalists do "face a hopeless task in explaining the complexities of science" [2].

## Why Help Journalists Overcome the Complexities of Science?

But why, beyond the benefit of the public good, should scientists take time out of their day to work with journalists? The answer is simple. Clear communication and greater awareness of your work can equal additional funding, enhanced career advancement and further scientific breakthroughs [3]. According to http://plainlanguage.gov, a recent study showed that medical articles reported in *The New England Journal of Medicine *and then reported in *The New York Times *receive about 73 percent more citations in medical reports than articles not reported in *The New York Times*. If a researcher is able to successfully communicate his or her points in *The New York Times*, chances are he or she also will be able to more clearly communicate the value and necessity if his or her work in a grant application. According to a National Science Foundation grant reviewer, the clearest and most succinct grant applications are usually the most compelling. If a scientist can pitch his or her grant proposal in three minutes or less, it has a better chance at being funded [4]. The same is true with the media. If you can communicate three or fewer compelling points about the results of your study, you are more likely to receive accurate and favorable coverage from journalists and the resulting greater awareness of your work.

Enhanced career opportunities also are a benefit to working with the media. Scientists who have good communications skills have a distinct advantage over their less communicative colleagues when they compete for prized positions. In addition, those scientists who are cited more and have greater "awareness" - not just about their research, but also about themselves in their fields - are more recognizable in their scientific community, and are likely to be sought after.

Finally, let's not overlook the fact that well-written articles that are picked up by the press help stimulate the "cross-fertilization" of research and ideas across broad disciplines, therefore improving the chances for even greater scientific breakthroughs.

## Tips for Working with the Media

So what should researchers keep in mind when working with the media? First, ask for help from the public affairs and/or media specialists within your organization. They are accustomed to working with the media on a regular basis and can help best prepare you for maximizing the media opportunity. Here are a few more tips:

• *Know Who You're Dealing With*. Many general consumer newsrooms are shrinking at a rapid pace and today's reporters are tasked with more responsibilities and fewer resources. Therefore, there is less time to interview credible professionals and fact-check - leaving greater potential for reporters and editors to get things wrong. Also, know that reporters are looking for stories and information that their readers/viewers/listeners will find interesting. So it is vital that you can quickly explain the results of your research and put it into context about its relevance. You must always be able to explain why the information is new and exciting, and compelling enough for a journalist to want to share that information with hundreds of thousands of people.

• *Communicate Simply and Clearly*. To have your work covered by the media, it is important to start with a well-written executive summary-style document, which outlines the key points of your findings. Federal government employees, through *The Plain Language Action and Information Network *(PLAIN), offer many tips for drafting user-friendly documents [5]. For instance, be sure to organize content to make it easy to understand with informative headings and subheadings, bold and italicize terms when appropriate, and use "plain language" such as writing in short, clear sentences with common, every day words, rather than industry jargon. For more tips, visit http://www.plainlanguage.gov. There you will also find training resources offered by *The Network *for researchers to take advantage of to improve communications skills.

Note, when discussing plain language usage, professionals are told that researchers often gravitate toward using technical language because that is what their peers "expect." However, it is argued that it is more effective to use language and formatting that is easy for everyone to understand and follow, no matter what the technical expertise. In doing so you will reap the benefits of clear communication and greater public awareness discussed earlier. A recent study by the *First Amendment Center *funded by the *Freedom Forum *also found this important in bridging the divide between journalists and researchers. One of the primary takeaways of the recent study is that it is important for scientists to work with publishers of scientific papers to include summaries of their findings- written in plain English and that puts the work into perspective and explains its relevance and importance up front [2].

• *Build Relationships*. Peer-reviewed, published articles have added credibility that journalists like, but they are not the only way to generate media interest and coverage. And, even if an article is published in a trade journal, there is a chance a mass-media journalist may miss it. Ask your public affairs/media specialists to help communicate your published work to journalists to educate the public about it. They can be helpful to you by generating news releases and other announcements about your work and distributing the information to the right people in the media. Also, be sure to get to know key reporters covering your field in the local and national media. Often times these people can be found attending major conferences and meetings. Introduce yourself. Briefly explain what you are working on and why it is important. Ask what the reporter is working on and see if you can be a resource to him or her.

Remember, most consumer reporters are often on very tight deadlines and must find expert resources for their stories quickly. Van Eperen & Company [6], is one of many strategic communications consulting firms that has relationships with consumer and health reporters and can help make this process go smoothly. Keep in mind that any and all responses are "on the record" and must be "quotable" - the key here again is to limit jargon and unnecessary words. Most important, the information you provide must be timely and accurate.

Beyond yourself, try to have one or two other colleagues in mind that are comfortable commenting on the subject and offer to put the reporter in touch with them. Journalists like to include quotes from at least two or three experts in stories to validate information and add varying perspectives. The more you know about the media, the more you will begin to realize that the best media spokespersons are subject matter experts, they're highly "quotable," and they readily make themselves available for interviews. If you meet these requirements, the more likely it is that you will be called back and included in future stories!

A 1997 study by the *Pew Research Center for the People and the Press *showed a fifth of Americans polled said they enjoyed stories about science and technology. That topic beat out religion, politics, international affairs, entertainment, consumer news, business and finance, famous people and culture and the arts. About the same time, a survey by the nation's largest newspaper chain, the Gannett Company, showed that 75 percent of readers were somewhat-to-very interested in science and technology [2]. So if the public appetite is there, then it is up to scientists and journalists to properly feed it.

## Potential Conflicts

LVE and JS belong to a company discussed in this manuscript and their article was solicited by FMM as an editorial.
